# Comparative Evaluation of the Effect of Different Post and Core Materials on Stress Distribution in Radicular Dentin by Three-Dimensional Finite Element Analysis

**Published:** 2018-03

**Authors:** Saied Nokar, Mehran Bahrami, Azam Sadat Mostafavi

**Affiliations:** 1 Associate Professor, Department of Prosthodontics, School of Dentistry, Tehran University of Medical Sciences, Tehran, Iran; 2 Assistant Professor, Department of Prosthodontics, School of Dentistry, Tehran University of Medical Sciences, Tehran, Iran

**Keywords:** Finite Element Analysis, Post and Core Technique, Dental Stress Analysis

## Abstract

**Objectives:**

The aim of this study was to investigate the stress distribution of different post and core materials in radicular dentin by three-dimensional finite element analysis (3D FEA).

**Materials and Methods:**

Twelve 3D models of a maxillary central incisor were simulated in the ANSYS 5.4 software program. The models were divided into three groups; the first group included: 1-Gold post and core and 2-Nickel-chromium (Ni-Cr) post and core restored with metal-ceramic restorations (MCRs). The second group included: 1-Stainless steel post, 2-Titanium post, 3-Carbon fiber post, 4-Glass fiber post, and 5-Quartz fiber post with composite cores and MCRs. The third group included: 1-Zirconia post and core, 2-Zirconia post, 3-Carbon fiber post, 4-Glass fiber post, and 5-Quartz fiber post; the last four models had composite cores restored with all-ceramic restorations (ACRs). Each specimen was subjected to a compressive load at a 45-degree angle relative to its longitudinal axis at a constant intensity of 100 N. The models were analyzed with regard to the stress distribution in dentin.

**Results:**

Two stress concentration sites were detected in the models. The first group showed the lowest stress levels in the cervical region, while the stress levels detected in the second group were higher than those in the first group and lower than those found in the third group. Fiber-reinforced posts induced a higher stress concentration between the middle and cervical thirds of the root compared to other posts.

**Conclusions:**

According to the results, since cast posts induce lower stresses in dentin, they are recommended for clinical use. Fiber-reinforced posts and ACRs caused the maximum stresses in dentin.

## INTRODUCTION

Restoration of endodontically treated teeth is challenging. Since the time Pierre Fauchard used gold, silver, or wooden dowels to retain crowns [1], various types of post-and-core systems have been introduced to dentistry. Endodontic posts may be cast with the core, such as gold and nickel-chromium (Ni-Cr) posts, or they may be prefabricated, such as titanium and stainless steel posts. Recently, non-metallic posts such as fiber-reinforced composite (FRC) and ceramic posts have been introduced as theoretically acceptable alternative materials [2–6]. One of the functions of post-and-core systems is to improve the tooth’s resistance by dispersing the functional forces along the root length. The material of a dental post is one of the factors affecting the stress distribution in dentin [7,8]. The stress distribution in post-and-core systems has been studied by many researchers using different theoretical or experimental techniques [7,8]. There are many contradictory viewpoints about the best choice of post material in the literature.

In some studies, a high-modulus root canal dowel has been recommended [9–13], while the others have advocated that the Young’s modulus (E) of a dowel should preferably be close to that of dentin [14–16]. Different in-vitro studies have determined the fracture resistance of the teeth restored with a dowel under static loading; however, their results are controversial. These studies have expressed a lower [17–20], the same [21–23], or a higher [2,24, 25] strength in the teeth restored with fiber dowels compared to those restored with metal dowels. One reason for this contradiction is that in-vitro studies are often unable to control several clinical variables. In a finite element analysis (FEA), Yaman et al [10] expressed that cast gold posts and cores yielded lower stress values than prefabricated stainless steel and titanium posts. Some studies pointed to a lower stress concentration in cast gold posts compared to FRC posts [26,27], while some others reported a lower stress concentration in FRC posts compared to metallic posts [4,5, 28]. Chen et al [16] expressed that polyethylene FRC posts did not significantly change the stress distribution compared to cast Ni-Cr posts.

Nonetheless, this is still a controversial subject. The aim of this study was to evaluate common post materials according to von Mises stress (VMS) and to report their effect on the stress distribution in radicular dentin by using three-dimensional (3D) FEA. According to the null hypothesis, there would be no significant statistical differences among the studied post materials with regard to the stress distribution in radicular dentin.

## MATERIALS AND METHODS

Twelve 3D models of a maxillary central incisor and its supporting structures were created by using the ANSYS 5.4 software program (Swanson Analysis System Inc., Houston, Texas, USA) to determine the stress distribution patterns in dentin.

**Fig. 1:**
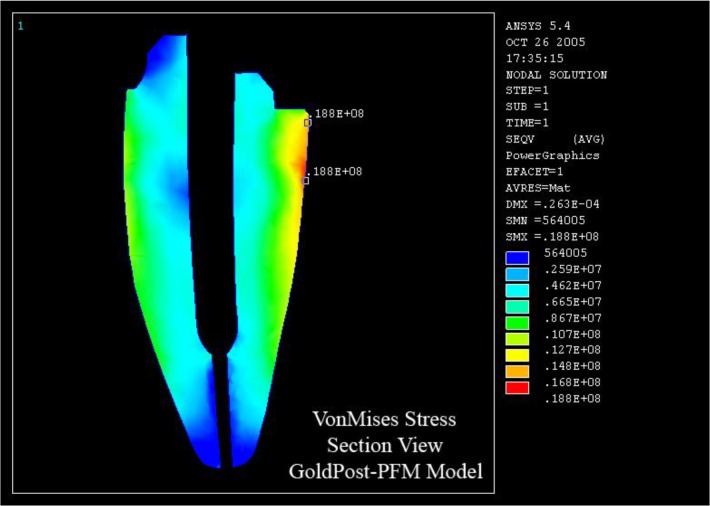
Classic tooth preparation for fabricating a cast post-and-core

The maxillary central incisor was selected because it is a single-rooted tooth with a relatively simple anatomy, and it is highly susceptible to fracture. The height of the remaining dentin was 1.5 mm to create a ferrule effect (Fig 1). In order to create a ferrule effect by the core, a 45-degree contra bevel was prepared around the vertical dentinal walls. In addition, 4 mm of gutta-percha was retained to preserve the apical seal.

The studied cores had a 9-mm length and a 4.7-mm diameter. The fabricated posts had a 1.7-mm diameter and a 9-mm length. Panavia F 2.0 resin cement (Kuraray America, Inc., New York, NY, USA) was used for cementing the cast post-and-core systems. The film thickness of the cement was considered to be 67μm.

**Fig. 2:**
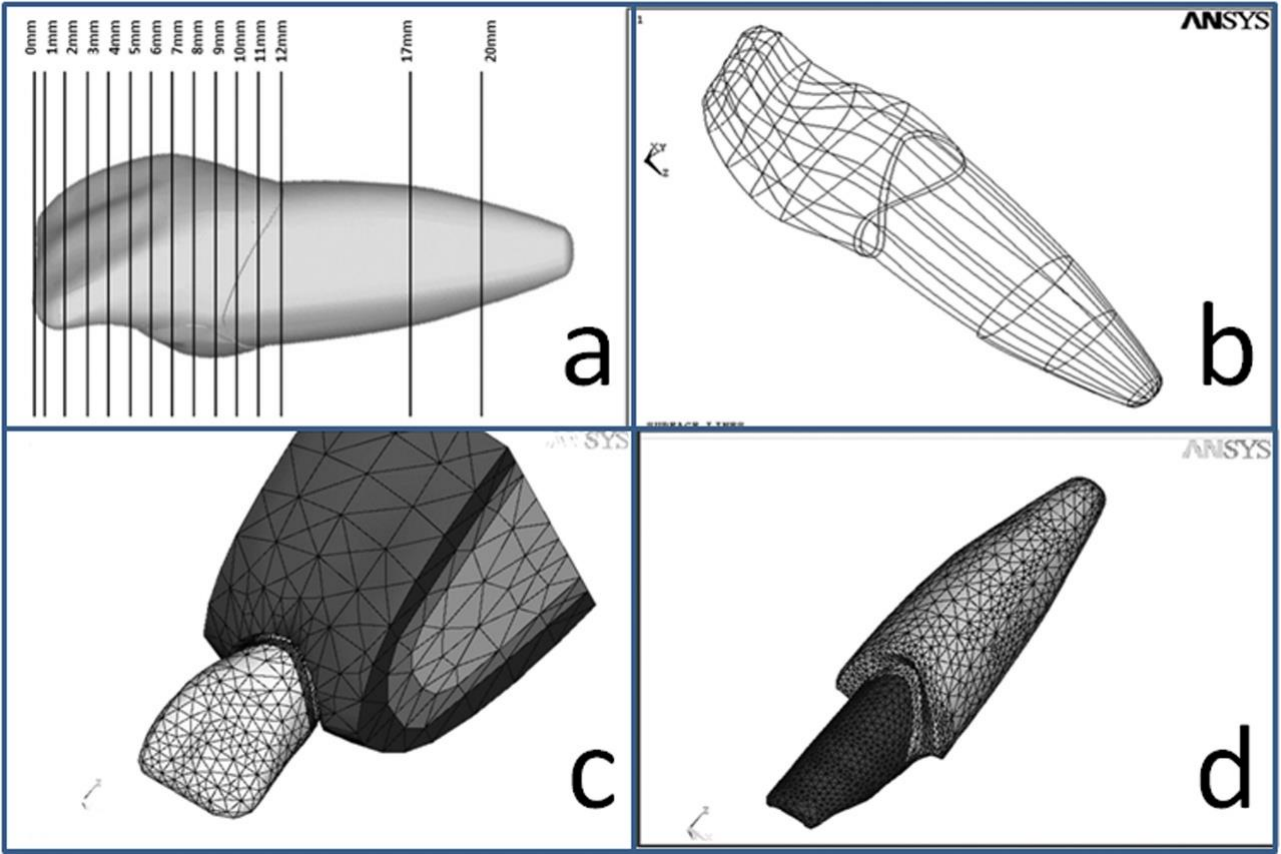
(a) Twelve cross-sections in the crown, and two cross-sections in the root. (b) The lines, surfaces, and volumes were designed. (c) and (d) The mesh was generated

The models were divided into three groups:

The first group included gold post-and-core and Ni-Cr post-and-core restored with metal-ceramic restorations (MCRs). The second group included stainless steel post, titanium post, carbon fiber post, glass fiber post, and quartz fiber post with composite cores restored with MCRs. The third group included zirconia post-and-core restored with all-ceramic restorations (ACRs), and zirconia post, carbon fiber post, glass fiber post, and quartz fiber post with composite cores and ACRs. The preferred finish line for the MCRs was the chamfer. For the ACRs, a radial shoulder finish line was prepared. All the crowns were considered to be cemented by using the same resin cement (Panavia F 2.0). The film thickness of the cement was considered to be 67μm. The “bottom-up method” was used to make the 3D models. In this research, anatomy-based geometric structures were considered for the enamel, dentin, pulp, porcelain or metal-ceramic crown, cortical bone, cancellous bone, the remaining root canal filling, and post. An acrylic model of the maxillary central incisor was fabricated three times greater than the real tooth size. This model was placed in a hexahedral box, and a transparent acrylic resin was poured around the tooth so that a polymeric hexahedral matrix was produced with an artificial tooth in the center. Twelve cross-sections were made in the crown (due to the fine details), while two cross-sections were made in the radicular part of the model (Fig. 2a). The images of these 14 cross-sections were transferred to the Adobe Photoshop software program (Adobe Systems Inc., San Jose, CA, USA) by using a scanner, and 14 key points were chosen on each cross-section. The position of each key point was determined according to the three intersecting coordinate planes of X, Y, and Z (Cartesian coordinates). The data related to each key point were transferred to the ANSYS software program, and a 3D image of the tooth was generated. Next, the lines, surfaces, and volumes were designed (Fig. 2b and 2c). The same method was used to create the inner parts of the model such as the post, cement, and gutta-percha. The gingiva, cancellous bone, cortical bone, periodontal ligament (PDL), lamina dura, and crown (metal-ceramic or all-ceramic) were also simulated for each model. All the materials, vital tissues, and continual interfaces between the materials were presumed elastic, homogenous, and isotropic. The mechanical properties (Young’s modulus and Poisson’s ratio) of each of the components used in this study are summarized in Table 1 [9,11,27,29–32]. During the meshing, the volumes were divided into smaller parts named elements. Each element consisted of eight nodes (a hexahedral element). The elements were connected to each other at their nodes (Fig. 2c and 2d). In this study, the finite element meshes were composed of nearly 4300 elements and 6000 nodes.

**Table 1.** Young’s modulus (E) and Poisson’s ratio (υ) of the materials in the present study

**Materials**	**Young's modulus (MN/m^2^)**	**Poisson’s ratio**
Enamel	41E9	0.31
Dentin	18.6E9	0.30
PDL	68.9E6	0.45
Cortical bone	13.7E9	0.30
Cancellous bone	1.37E9	0.30
Gingiva	19.06E6	0.30
Gutta-percha	0.69E6	0.45
Porcelain	69E9	0.28
Stainless steel post	200E9	0.33
Gold post	88E9	0.35
Gold alloy coping	77E9	0.35
Quartz fiber	18.7E9	0.30
Carbon fiber	21E9	0.31
Glass fiber	40E9	0.26
Zirconia	200E9	0.33
Ni-Cr	200E9	0.33
Titanium	112E9	0.33
Composite core	12E9	0.30
IPS Empress II	96E9	0.25
Resin cement	18.6E9	0.28

PDL=Periodontal ligament, Ni-Cr=Nickel-Chromium

**Fig. 3:**
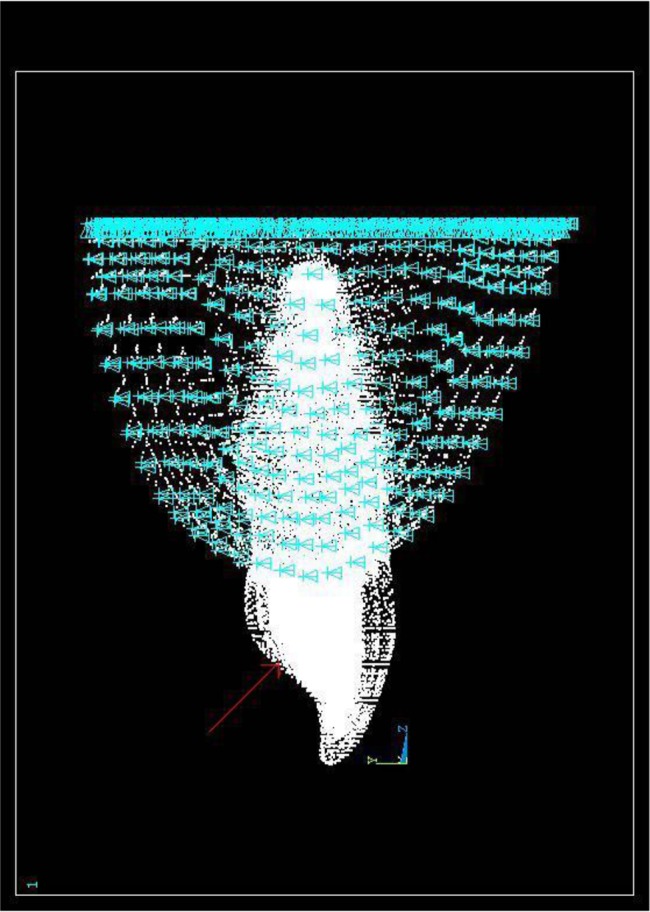
A 100-N compressive load was applied to a load-bearing area of 1 mm^2^ on the lingual surface of the tooth

A compressive load with a constant intensity of 100 N was applied to a load-bearing area of 1 mm^2^ on the lingual surface of the tooth at an angle of 45 degrees relative to the longitudinal axis of the tooth in order to simulate a centric occlusal contact with the opposite tooth (Fig 3). Finally, the models were analyzed with regard to the stress distribution in dentin.

## RESULTS

In this FEA, VMS (equivalent stress) was considered because it has a higher validity than stress analysis. This parameter is shown by δe and is obtained from the following formula:

δe=(1/2[(δ1−δ2)2+(δ2−δ3)2+(δ3−δ1)2])12

δ1, δ2, and δ3 are the principal stress components. The VMS shows the location of the maximum stress without determining its nature (either tensile or compressive) [33]; therefore, it is useful in the experiments which only determine the existence of stress, similar to the current study.

In all the models, stress concentration was detected at two areas of the root:

1. The junction of the middle and cervical thirds of the root.
2. The cervical part of the root.

The results are presented as the maximum VMS values in Table 2. Of course, The VMS is present in all the components, but only radicular dentin stresses are reported in this study. A convenient way of reporting the VMS is a color representation of the stress distribution.

**Table 2.** Maximum von Mises stress (VMS) in the models

**Models**			**Max VMS (MPa)**	

Post	Core	Crown	At the gingival border	between the middle and cervical thirds of the root
Gold	Gold	MCR	18.8	18.8
Ni-Cr	Ni-Cr	MCR	18	18.1
Stainless steel	Composite	MCR	20.5	18.4
Titanium	Composite	MCR	20.3	18.6
Carbon fiber	Composite	MCR	20.9	19.1
Glass fiber	Composite	MCR	21.5	19.5
Quartz fiber	Composite	MCR	21	19.5
Zirconia	Zirconia	ACR	22.7	18.2
Zirconia	Composite	ACR	23.4	18.1
Carbon fiber	Composite	ACR	24.5	19.5
Glass fiber	Composite	ACR	24.4	19.2
Quartz fiber	Composite	ACR	24.5	19.6

Ni-Cr=Nickel-Chromium, MCR=Metal-Ceramic Restoration, ACR=All-Ceramic Restoration

## DISCUSSION

In the current 3D FEA, the VMS of common post materials was evaluated. The null hypothesis was rejected. According to the results, in all the models, two stress concentration regions were identified: 1) the cervical region of the root, which was covered with the cervical edges of the crown, and 2) between the middle and cervical thirds of the root, where the cortical bone comes to an end on the root. In both regions, compressive stresses concentrated on the buccal side, while tensile stresses concentrated on the palatal side of the studied models. Several studies have reported the cervical region of the root as a stress concentration site [4,11,12,34,35]. Assif and Gorfil [35] stated that this area is the interface between materials with different Young’s modulus values.

**Fig. 4:**
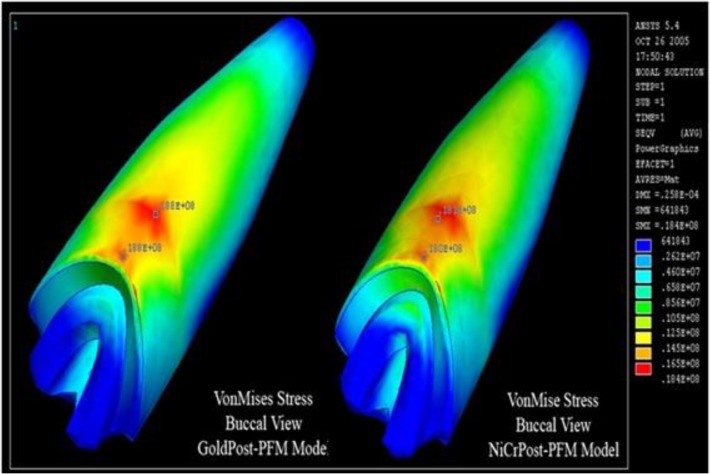
Maximum von Mises stress (VMS) in gold post-and-core (GoldPost-MCR) and nickel-chromium (Ni-Cr) post-and-core (NiCrPost-MCR) restored with metal-ceramic restorations (MCRs)

In the first group, stress levels in each model were similar at both stress concentration regions. However, Ni-Cr posts showed lower stress levels compared to gold posts (Fig 4).

**Fig. 5:**
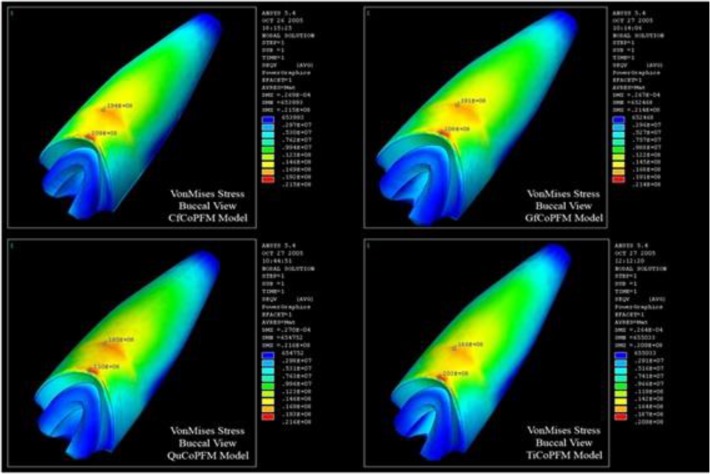
Maximum von Mises stress (VMS) in carbon fiber post (CfCoMCR), glass fiber post (GfCoMCR), quartz fiber post (QuCoMCR), and titanium post (TiCoMCR) with composite cores and metal-ceramic restorations (MCRs)

In the second group, stainless steel and titanium posts showed lower stresses in dentin compared to FRC posts. In all the five models of this group, the VMS values in the cervical region of the root were higher than those between the middle and cervical thirds of the root (Fig. 5). Yang et al [36] reported the same results.

**Fig. 6:**
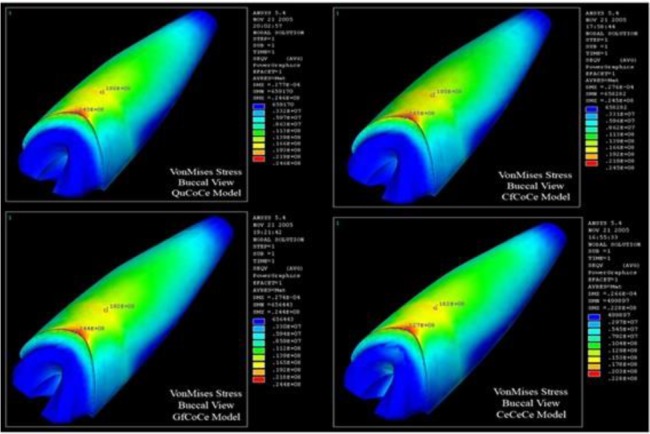
Maximum von Mises stress (VMS) in quartz fiber post (QuCoCe), carbon fiber post (CfCoCe), glass fiber post (GfCoCe), and zirconia post-and-core (CeCeCe) restored with all-ceramic restorations (ACRs)

In the third group, cervical stresses were higher compared to the other groups. The least amount of stress was detected in the model with a zirconia post-and-core (Fig. 6); this has also been confirmed by similar studies [10,11, 34]. Assmusen et al [11] and Toksavul et al [34] reported lower stress levels for zirconia post-and-core compared to titanium posts. Therefore, zirconia post-and-core systems may be an alternative to metallic posts.

According to the results of the present study, cast post-and-core systems showed a more favorable stress distribution pattern as they induced a lower VMS in radicular dentin, especially in the cervical region of the root. They also induced almost the same stress levels in both stress concentration areas. Among the prefabricated post models, stainless steel, titanium, and zirconia posts demonstrated nearly the same stress levels between the middle and cervical thirds of the root. However, these three posts showed lower levels of VMS between the middle and cervical thirds of the root in comparison with FRC posts (Fig. 6).

Cervical stresses in the models restored with ACRs were significantly higher than those in the models restored with MCRs because IPS Empress II is stiffer than MCR [28,37]. This finding was similar to the results of the studies by Pegoretti et al [28] and Eskitaşcioğlu et al [37].

In the present research, the zirconia post-and-core system showed a more favorable stress distribution pattern than the zirconia post-composite core, which confirms the results found by Heydecke et al [38] and Butz et al [39].

Cast metal post-and-core systems caused lower levels of stress compared to prefabricated metallic posts, similar to the results of the study by Yaman et al [40]. In addition, the findings of the current study confirmed that an increase in the Young’s modulus of the dowels reduces dentinal stresses. Some FEA studies have reported that FRC posts cause less stress than metallic posts [4,5,28,35].

Different mechanical properties (especially for FRCs), different modeling techniques, use of 2D or 3D FEA, different forces and directions of load application are some of the factors which may have affected the results of these studies.

Most mechanical experiments have recorded a higher fracture threshold for metallic posts compared to FRC posts; however, it has also been explained that prefabricated FRC posts show more favorable fractures in comparison with metallic posts [9,17,19,40,41].

Ferrari et al [6] reported that the teeth restored with carbon FRC posts had a significantly higher survival rate after 4 years than the teeth restored with metal posts. In a clinical trial designed by King et al [42], the teeth restored with carbon FRC posts did not perform as well as conventional wrought precious alloy posts. However, clinical studies comparing fiber dowels with metal dowels are scarce. Heydecke et al [38] and Butz et al [39] reported that zirconia post-and-core systems could be used as an alternative to metal posts; however, the survival rate of zirconia posts/composite cores was lower than that of cast posts. In the study by Dilmener et al [43], cast metal post-and-core systems were found to be more fracture resistant than zirconia posts/composite cores. According to Fraga et al [44], cast Ni-Cr post-and-core systems showed a significantly higher resistance to fracture than prefabricated stainless steel posts. Dilmener et al [43] found the same results. In a research by Barjau Escribano et al [29], a significantly lower failure load was found for the teeth restored with stainless steel posts compared to those restored with glass fiber posts. According to Asmussen et al [11] and Toksavul et al [34], zirconia ceramic posts created less stress concentration in dentin than glass FRC and titanium posts. In the present study, cast metal posts showed a more favorable stress distribution pattern than the other posts. Nevertheless, supplementary clinical studies are required to further evaluate the properties of FRC posts.

## CONCLUSION

Within the limitations of this theoretical FEA, the following conclusions were drawn:

1. In all the models, two sites of stress concentration appeared: at the cervical edge of the root, and between the middle and cervical thirds of the root.
2. Metallic cast posts showed the least amount of stress concentration.
3. The models reconstructed with MCRs showed higher stresses in the cervical region of the root. These stresses increased in the models restored with ACRs.
4. Stainless steel, titanium, and ceramic posts induced a more favorable stress distribution pattern in comparison with FRC posts.
5. Among the models, FRC posts showed higher stress levels in the area between the middle and cervical thirds of the root.

The findings of the current study may help the clinicians to select the most suitable post-and-core systems according to the clinical status of each tooth. Of course, additional clinical investigations are required to verify these theoretical in-vitro results.

## References

[1-] Ingle JI Taintor JFJ Endodontics. 3rd ed Lea & Febiger, Philadelphia, 1985:46–52.

[2-] Akkayan B Gülmez T Resistance to fracture of endodontically treated teeth restored with different post systems. J Prosthet Dent. 2002 4;87(4):431–7. 12011860 10.1067/mpr.2002.123227

[3-] Zhang Y Lu Z Wang K [Fracture strength of custom-fabricated celay all-ceramic post and core]. [Article in Chinese]. Hua Xi Kou Qiang Yi Xue Za Zhi. 2002 2;20(1):39–41, 44. 12593200

[4-] de Castro Albuquerque R Polleto LT Fontana RH Cimini CA Stress analysis of an upper central incisor restored with different posts. J Oral Rehabil. 2003 9;30(9):936–43. 12950976 10.1046/j.1365-2842.2003.01154.x

[5-] Lanza A Aversa R Rengo S Apicella D Apicella A 3D FEA of cemented steel, glass and carbon posts in a maxillary incisor. Dent Mater. 2005 8;21(8):709–15. 16026666 10.1016/j.dental.2004.09.010

[6-] Ferrari M Vichi A Garcia-Godoy F Clinical evaluation of fiber-reinforced epoxy resin posts and cast post and cores. Am J Dent. 2000 5;13(Spec No):15B–18B. 11763866

[7-] Hassan-Ahangari A Geramy A Valian A Ferrule Designs and Stress Distribution in Endodontically Treated Upper Central Incisors: 3D Finite Element Analysis. J Dent (Tehran). 2008 2;5(3):105–110.

[8-] Geramy A Eghbal MJ Ehsani S Stress distribution changes after root canal therapy in canine model: a finite element study. Iran Endod J. 2008 Fall;3(4):113–8. 24082903 PMC3782244

[9-] Asmussen E Peutzfeldt A Heitmann T Stiffness, elastic limit, and strength of newer types of endodontic posts. J Dent. 1999 5;27(4):275–8. 10193104 10.1016/s0300-5712(98)00066-9

[10-] Yaman SD Karacaer O Sahin M Stress distribution of post-core applications in maxillary central incisors. J Biomater Appl. 2004 1;18(3):163–77. 14871043 10.1177/0885328204034745

[11-] Asmussen E Peutzfeldt A Sahafi A Finite element analysis of stresses in endodontically treated, dowel-restored teeth. J Prosthet Dent. 2005 10;94(4):321–9. 16198168 10.1016/j.prosdent.2005.07.003

[12-] Ho MH Lee SY Chen HH Lee MC Three-dimensional finite element analysis of the effects of posts on stress distribution in dentin. J Prosthet Dent. 1994 10;72(4):367–72. 7990041 10.1016/0022-3913(94)90555-x

[13-] Cailleteau JG Rieger MR Akin JE A comparison of intracanal stresses in a post-restored tooth utilizing the finite element method. J Endod. 1992 11;18(11):540–4. 1298790 10.1016/S0099-2399(06)81210-0

[14-] Assif D Oren E Marshak BL Aviv I Photoelastic analysis of stress transfer by endodontically treated teeth to the supporting structure using different restorative techniques. J Prosthet Dent. 1989 5;61(5):535–43. 2664139 10.1016/0022-3913(89)90272-2

[15-] Isidor F Odman P Brøndum K Intermittent loading of teeth restored using prefabricated carbon fiber posts. Int J Prosthodont. 1996 Mar-Apr;9(2):131–6. 8639235

[16-] Chen XT Li XN Guan ZQ Liu XG Gu YX [Effects of post material on stress distribution in dentine]. [Article in Chinese]. Zhonghua Kou Qiang Yi Xue Za Zhi. 2004 7;39(4):302–5. 15454015

[17-] Fokkinga WA Kreulen CM Vallittu PK Creugers NH A structured analysis of in vitro failure loads and failure modes of fiber, metal and ceramic post-and-core systems. Int J Prosthodont. 2004 Jul-Aug;17(4):476–82. 15382786

[18-] Martínez-Insua A da Silva L Rilo B Santana U Comparison of the fracture resistances of pulpless teeth restored with a cast post and core or carbon fiber post with a composite core. J Prosthet Dent. 1998 11;80(5):527–32. 9813801 10.1016/s0022-3913(98)70027-7

[19-] Sirimai S Riis DN Morgano SM An in vitro study of the fracture resistance and the incidence of vertical root fracture of pulpless teeth restored with six post-and-core systems. J Prosthet Dent. 1999 3;81(3):262–9. 10050112 10.1016/s0022-3913(99)70267-2

[20-] Sidoli GE King PA Setchell DJ An in vitro evaluation of a carbon fiber-based post and core system. J Prosthet Dent. 1997 7;78(1):5–9. 9237139 10.1016/s0022-3913(97)70080-5

[21-] Drummond JL In vitro evaluation of endodontic posts. Am J Dent. 2000 5;13(Spec No):5B–8B. 11763868

[22-] Raygot CG Chai J Jameson DL Fracture resistance and primary failure mode of endodontically treated teeth restored with a carbon fiber-reinforced resin post sys-tem in vitro. Int J Prosthodont. 2001 Mar-Apr;14(2):141–5. 11843450

[23-] McDonald AV King PA Setchell DJ In vitro study to compare impact fracture resistance of intact root-treated teeth. Int Endod J. 1990 11;23(6):304–12. 2098347 10.1111/j.1365-2591.1990.tb00110.x

[24-] King PA Setchell DJ An in vitro evaluation of a prototype CFRC prefabricated post developed for the restoration of pulpless teeth. J Oral Rehabil. 1990 11;17(6):599–609. 2283555 10.1111/j.1365-2842.1990.tb01431.x

[25-] Duret B Duret F Reynaud M Long-life physical property preservation and post endodontic rehabilitation with the Composipost. Compend Contin Educ Dent Suppl. 1996 ;(Suppl 20):S50–6. 12089762

[26-] Yang HS Lang LA Molina A Felton DA The effects of dowel design and load direction on dowel-and-core restorations. J Prosthet Dent. 2001 6;85(6):558–67. 11404756 10.1067/mpr.2001.115504

[27-] Pierrisnard L Bohin F Renault P Barquins M Corono-radicular reconstruction of pulpless teeth: A mechanical study using finite element analysis. J Prosthet Dent. 2002 10;88(4):442–8. 12447223 10.1067/mpr.2002.128376

[28-] Pegoretti A Fambri L Zappini G Bianchetti M Finite element analysis of a glass fiber reinforced composite endodontic post. Biomaterials. 2002 7;23(13):2667–2682. 12059016 10.1016/s0142-9612(01)00407-0

[29-] Barjau Escribano A Sancho Bru JL Forner Navarro L Rodríguez Cervantes PJ Pérez Gónzález A Sánchez Marín FT Influence of prefabricated post material on restored teeth: fracture strength and stress distribution. Oper Dent. 2006 Jan-Feb;31(1):47–54. 16536193 10.2341/04-169

[30-] Ko CC Chu CS Chuag KH Lee MC Effects of posts on dentin stress distribution in pulpless teeth. J Prosthet Dent. 1992 9;68(3):421–7. 1432755 10.1016/0022-3913(92)90404-x

[31-] Reinhardt RA Krejci RF Pao YC Stannard JG Dentin stresses in post-reconstructed teeth with diminishing bone support. J Dent Res. 1983 9;62(9): 1002–8. 6348112 10.1177/00220345830620090101

[32-] Holmes DC Diaz-Arnold AM Leary JM Influence of post dimension on stress distribution in dentin. J Prosthet Dent. 1996 2;75(2):140–7. 8667271 10.1016/s0022-3913(96)90090-6

[33-] Digital Engineering Stress in FEA: Part 3. Available at: http://www.digitaleng.news/de/stress-in-fea-part-3/ Accessed July 1, 2016.

[34-] Toksavul S Zor M Toman M Güngör MA Nergiz I Artunç C Analysis of dentin-al stress distribution of maxillary central incisors subjected to various post-and-core applications. Oper Dent. 2006 Jan-Feb;31(1):89–96. 16536199 10.2341/04-192

[35-] Assif D Gorfil C Biomechanical considerations in restoring endodontically treated teeth. J Prosthet Dent. 1994 6;71(6):565–7. 8040817 10.1016/0022-3913(94)90438-3

[36-] Yang HS Lang LA Guckes AD Felton DA The effect of thermal change on various dowel-and-core restorative materials. J Prosthet Dent. 2001 7;86(1): 74–80. 11458265 10.1067/mpr.2001.115503

[37-] Eskitaşcioğlu G Belli S Kalkan M Evaluation of two post core systems using two different methods (fracture strength test and a finite elemental stress analysis). J Endod. 2002 9;28(9):629–33. 12236304 10.1097/00004770-200209000-00001

[38-] Heydecke G Butz F Hussein A Strub JR Fracture strength after dynamic loading of endodontically treated teeth restored with different post-and-core systems. J Prosthet Dent. 2002 4;87(4):438–45. 12011861 10.1067/mpr.2002.123849

[39-] Butz F Lennon AM Heydecke G Strub JR Survival rate and fracture strength of endodontically treated maxillary incisors with moderate defects restored with different post-and-core systems: an in vitro study. Int J Prosthodont. 2001 Jan-Feb;14(1):58–64. 11842907

[40-] Yaman SD Alaçam T Yaman Y Analysis of stress distribution in a maxillary central incisor subjected to various post and core applications. J Endod. 1998 2;24(2):107–11. 9641141 10.1016/S0099-2399(98)80087-3

[41-] Bolhuis P de Gee A Feilzer A Influence of fatigue loading on four post-and-core systems in maxillary premolars. Quintessence Int. 2004 9;35(8):657–67. 15366533

[42-] King PA Setchell DJ Rees JS Clinical evaluation of a carbon fiber reinforced carbon endodontic post. J Oral Rehabil. 2003 8;30(8):785–9. 12880400 10.1046/j.1365-2842.2003.01178.x

[43-] Dilmener FT Sipahi C Dalkiz M Resistance of three new esthetic post-and-core systems to compressive loading. J Prosthet Dent. 2006 2;95(2):130–6. 16473087 10.1016/j.prosdent.2005.11.013

[44-] Fraga RC Chaves BT Mello GS Siqueira JF Jr Fracture resistance of endodontically treated roots after restoration. J Oral Rehabil. 1998 11;25(11):809–13. 9846900 10.1046/j.1365-2842.1998.00327.x

